# Meningitis and Radiculomyelitis Caused by *Angiostrongylus cantonensis*

**DOI:** 10.3201/eid1506.081263

**Published:** 2009-06

**Authors:** Tomislav Maretić, Marta Perović, Adriana Vince, Davorka Lukas, Paron Dekumyoy, Josip Begovac

**Affiliations:** University Hospital for Infectious Diseases Dr Fran Mihaljevic, Zagreb, Croatia (T. Maretić, M. Perović, A. Vince, D. Lukas, J. Begovac); Mahidol University, Bangkok, Thailand (P. Dekumyoy)

**Keywords:** Angiostrongylus cantonensis, eosinophilic meningitis, lumbosacral myeloradiculopathy Elsberg syndrome, parasites, letter

**To the Editor:** *Angiostrongylus cantonensis* infection is endemic in regions such as Southeast Asia, China, the Pacific Basin, and the Caribbean, but international travel has spread the disease elsewhere, including Europe (*1*–*10*). Dissemination of the parasite to many regions has also occurred because of the ship-borne international migration of rats and the diversity of potential intermediate hosts. The target organ in humans is the central nervous system in which an eosinophilic reaction develops in response to dying larvae. We report a case of eosinophilic meningitis and lumbosacral myeloradiculopathy caused by *A. cantonensis* and present a review of cases of *A. cantonensis* infections from Europe.

A 47-year-old merchant seaman was admitted to the University Hospital of Infectious Diseases, Zagreb, Croatia, in March, 2006 on the 17th day of illness because of fever, headache, vomiting, and constipation. At the end of the first week of illness, paresthesias developed in his feet; on the 10th day of illness, he also noticed difficulties with urination. He had returned from a 1-month trip to Southeast Asia (Malaysia and Singapore) 35 days before the onset of symptoms and recalled eating vegetables and salads. He also consumed shrimp, but he believed that they were from salt water. On physical examination, we noticed increased muscle tone, tremor of the tongue and upper limbs, and decreased deep tendon reflexes of the lower limbs. He experienced urinary retention, and catheterization was required. Saddle anesthesia was observed. There was no neck stiffness, and the results of the rest of the physical examination were normal.

His blood leukocyte count was 11.5 × 10^9^/L with 80% neutrophils, 12% lymphocytes, 4% monocytes, 2% basophils, and 2% eosinophils. Cerebrospinal fluid (CSF) analysis showed 320 cells/μL with 6.5% eosinophils (21 eosinophils/μL). Results of CSF testing by PCR for herpes simplex virus 1 (HSV-1) and HSV-2 DNA were negative, as were cultures for bacteria, mycobacteria, and fungi. Results of serum and CSF antibody tests for *Borrelia burgdorferi*, *Treponema pallidum*, HSV-1, HSV-2, tick-borne encephalitis virus, *Toxoplasma gondii*, *Taenia solium, Toxocara* spp., and *Trichinella* spp. were also negative. Results of stool examination for *Ascaris lumbricoides*, *Trichuris trichiura, Taenia* spp., *Giardia intestinalis*, *Strongyloides* spp., and *Entamoeba histolytica* were negative. The patient was also negative for HIV by ELISA. Magnetic resonance imaging scans of the brain and spine were unremarkable. *A.* *cantonensis* infection was diagnosed by immunoblot testing at the Department of Helminthology, Faculty of Tropical Medicine, Mahidol University, Bangkok. Antibodies against *A. cantonensis* 31-kDa antigen were detected in serum and CSF of the patient; antibodies against *Gnathostoma spinigerum* were not detected.

Treatment was symptomatic; to lessen the headache, 4 lumbar punctures were performed. After 1 month, the patient’s general condition was greatly improved; however, minor symptoms such as diminished concentration, slow thinking, and mild headache persisted. Urinary retention lasted for 38 days, and the patient had occasional mild headaches and paresthesia in his feet for the next 5 months.

Because large numbers of persons from Europe travel to destinations where angiostrongyliasis is endemic, it is somewhat surprising that the infection has been rarely described in Europe. In a Google and Medline Internet literature search, we identified 9 additional case reports and 1 report on a cluster of 5 *A. cantonensis* infections. The first report was in a 14-month-old child born in Tahiti who became ill in France in 1988 (*1*). Eight cases, as did our case, involved travelers returning to Europe after a visit to disease-endemic areas (*2*–*9*) (Table). Not all cases were serologically confirmed, most likely because antibody tests for *A. cantonensis* infection have not been widely available. In a retrospective cohort study, 5 French policemen, who returned from French Polynesia with severe headache and blood eosinophilia, were believed to have eosinophilic meningitis caused by *A. cantonensis* (*10*). However, CSF examination was performed in only 1 patient, and none of the cases were serologically confirmed.

**Table:** Epidemiologic and clinical findings from 11 reported case-patients with *Angiostrongylus cantonensis* infection, Europe*

Country, year of report	Patient age, y/ sex	Possible country of origin	Suspected food	Clinical features	Diagnosis	Treatment	Ref.
France, 1988	1/F	Tahiti	Not reported	Eosinophillic meningoencephalitis, lumbosacral myeloradiculitis, tetraplegia coma, hypertensive hydrocephalus, paresthesias	IFA	Thiabendazole, steroids, ventriculoperitoneal catheter	(*1*)
Switzerland, 1995	46/F	Tahiti	Freshwater shrimp	Eosinophilic meningoradiculits	Clinical	Supportive	(*2*)
France, 1996	25/F	Tahiti	Raw fish	Eosinophilic meningitis, paresthesias (left lower leg)	Clinical	Supportive	(*3*)
France, 2002	16/M	Tahiti	Not reported	Eosinophilic meningoencephalitis, cranial palsy (n. abducens), cerebellar syndrome, paresthesias	IFA	Ivermectin	(*4*)
Switzerland, 2004	26/M	Cuba	Not reported	Eosinophilic meningitis, generalized hyperesthesias	WB	Supportive	(*5*)
Germany, 2006	27/F	Dominican Republic	Not reported	Eosinophilic meningitis, paresthesias (right elbow, right thigh)	WB	Albendazole, steroids	(*6*)
Italy, 2007	30/M	Dominican Republic	Freshwater shrimp	Eosinophilic meningitis, generalized paresthesias	Clinical	Mebendazole,† steroids	(*7*)
UK, 2007	30/F	Thailand	Snails	Eosinophilic meningitis, cranial nerve palsy (right n. abducens), altered sensation (lateral border of right leg)	Serologic‡	Supportive	(*8*)
Belgium, 2008	22/F	Costa Rica, Ecuador, Chile, Argentina, Fiji Islands	Sashimi, ceviche (raw fish), salads	Eosinophilic meningitis, paresthesias (left hemithorax, feet)	WB	Albendazole, steroids	(*9*)
France, 2008§	26–36/M	French Polynesia	Uncooked freshwater prawns	Eosinophilic meningitis¶	Clinical	Ivermectin (1 patient) or albendazole; steroids (1 patient)	(*10*)
Croatia	47/M	Malaysia, Singapore	Vegetables, salads, shrimp#	Eosinophilic meningitis, lumbosacral myeloradiculitis (conus medullaris syndrome), generalized paresthesias	WB	Supportive (repeated lumbar puncture)	This study

*Ref., reference; IFA, immunofluorescent antibody assay; WB, Western blot. †One dose only. ‡Method not specified. §Five French policemen. ¶Diagnosis confirmed by cerebrospinal fluid analysis in only 1 patient. #The patient thought he consumed saltwater shrimp but was uncertain.

Definitive diagnosis of angiostrongyliasis would require identification of larvae or young adults in human tissue, such as the brain, CSF, and eye chamber, which is rarely achieved. Thus, the diagnosis is usually made on the basis of serologic test results. Specific *A. cantonensis* antigens (29 kDa, 31 kDa, and 32 kDa) were identified; antibodies against these antigens can be detected by ELISA, dot-blot ELISA, or Western blot.

In most patients, *A.* *cantonensis* causes a benign and self-limiting disease; treatment is usually symptomatic. Data are limited, but mostly favorable, on the use of steroids, albendazole, and mebendazole. However, the administration of antihelmintics without steroids is not recommended because such treatment might elicit deleterious inflammatory responses to dying worms within the nervous system or ocular structures.

In summary, the presence of headache, fever, and paresthesias in travelers returning from disease-endemic areas should alert clinicians to the possibility of eosinophilic meningitis caused by *A. cantonensis*. With growing international travel, physicians may encounter *A. cantonensis* infection more frequently.
